# The identity of the Sri Lankan *Amblypharyngodon* (Teleostei, Cyprinidae)

**DOI:** 10.3897/zookeys.820.29632

**Published:** 2019-01-28

**Authors:** Hiranya Sudasinghe, Rohan Pethiyagoda, Kalana Maduwage, Madhava Meegaskumbura

**Affiliations:** 1 Evolutionary Ecology and Systematics Lab, Department of Molecular Biology and Biotechnology, University of Peradeniya, Peradeniya, Sri Lanka; 2 Postgraduate Institute of Science, Faculty of Science, University of Peradeniya, Sri Lanka; 3 Ichthyology Section, Australian Museum, 6 College Street, Sydney, NSW 2010, Australia; 4 Department of Biochemistry, Faculty of Medicine, University of Peradeniya, Sri Lanka; 5 Guangxi Key Laboratory of Forest Ecology & Conservation, College of Forestry, Guangxi University, Nanning, China

**Keywords:** *
Amblypharyngodon grandisquamis
*, *
Amblypharyngodon melettinus
*, biodiversity hotspot, DNA barcoding, integrative taxonomy, island endemism

## Abstract

Morphological and molecular analyses of specimens representative of the geographic range of the cyprinid genus *Amblypharyngodon* in Sri Lanka suggest the presence of only a single species in the island, for which the name *Amblypharyngodongrandisquamis* Jordan & Starks, 1917, is available. *Amblypharyngodongrandisquamis* is a species endemic to Sri Lanka, distributed across the lowlands of both of the island’s main climatic zones. It is distinguished from all other species of *Amblypharyngodon*, including the three species recorded from peninsular India (*A.mola*, *A.microlepis*, and *A.melettinus*), by a suite of characters that includes a body depth of 26.9–31.2% of the standard length (SL), 42–56 scales in the lateral series (of which usually 8–16 are pored), 20–24 circumpeduncular scales, 14–17 scale rows between the origins of the dorsal and pelvic fins, a dorsal-fin height of 21.1–27.6% SL, 18–19 caudal vertebrae and an eye diameter of 22.7–30.5% of the head length. *Amblypharyngodongrandisquamis* differs from *A.melettinus* and *A.mola* by uncorrected pairwise genetic distances of more than 9% and 6%, respectively, for the mitochondrial cytochrome oxidase subunit 1 (COI) gene.

## Introduction

The cyprinid genus *Amblypharyngodon* Bleeker, which has a range extending from the Indian subcontinent to Southeast Asia, is considered to contain just five valid species: *A.atkinsonii* (Blyth), *A.chulabhornae* Vidthayanon & Kottelat, *A.melettinus* (Valenciennes), *A.microlepis* (Bleeker), and *A.mola* (Hamilton) (Vidthayanon and Kottelat 1990). The genus is characterised by having pharyngeal teeth that are compressed, with the crown blunt and enlarged; the lateral line incomplete, with 42–79 small scales in the lateral series; seven branched dorsal-fin rays; a small maximum size (standard length, SL), ranging from approximately 40 to 150 mm; and the absence of an upper lip, barbels, and fleshy labial folds (Vidthayanon and Kottelat 1990).

The genus was first reported from Sri Lanka by Günther (1868: 202), who identified specimens from the island as *Leuciscusmelettina* Valenciennes, in Cuvier and Valenciennes 1844 (hereafter Valenciennes 1844), type locality Bombay [Mumbai], India. Jordan and Starks (1917) described a second species from Sri Lanka, *A.grandisquamis* (type locality “river at Colombo”), which they distinguished from *A.melettinus* as follows: “This species is closest to *Amblypharyngodonmelettinus*, but is deeper and has much larger scales”. Since then, the identity of the species of *Amblypharyngodon* inhabiting Sri Lanka has been confused, with some authors considering only *A.melettinus* to be present in the island (e.g., Deraniyagala 1952: 45, Senanayake 1980: 167, Pethiyagoda 1991: 25), while others consider both *A.melettinus* and *A.grandisquamis* to be present (Pethiyagoda 2006, MOE 2012, De Silva et al. 2015). No specimen-based study has been conducted to resolve this confusion until now.

Here we review Sri Lankan *Amblypharyngodon* using an integrative taxonomic approach (Dayrat 2005) that combines morphological and molecular data, and show that only a single species is present in the island, for which the name *A.grandisquamis* Jordan & Starks, 1917 is available.

## Materials and methods

### Metrics and meristics

Measurements and counts follow Sudasinghe et al. (2018a) except that the lateral-line scale count is given as the number of pored lateral-line scales + the scales between the last pored scale and the base of the hypural plate. In addition, the pelvic-anal distance was measured between the origins of the pelvic and anal fins, respectively. All counts and measurements were taken on the left side of specimens whenever possible. Body measurements and head length are given as proportions of standard length; and subunits of the head as proportions of head length. Values in parentheses following a count indicate the frequency of that count. Vertebral counts and osteological descriptions are based on cleared and stained specimens following the single-staining method of Taylor and Van Dyke (1985). Osteological descriptions follow Vidthayanon and Kottelat (1990) and Conway (2011).

Comparative data for *A.chulabhornae* and (in part) *A.atkinsonii* and *A.microlepis* are from Vidthayanon and Kottelat (1990).

### Material

Specimens referred to in the text are deposited in the collections of Muséum national d’Histoire naturelle, Paris, France (**MNHN**); Stanford University, Palo Alto, California, USA. Some specimens are on long term loan to the California Academy of Sciences, San Francisco (**CAS-SU**); Field Museum of Natural History, Chicago, USA (**FMNH**); Australian Museum, Sydney (**AMS**); the Wildlife Heritage Trust of Sri Lanka (**WHT**), now at the National Museum of Sri Lanka; Bombay Natural History Society, Mumbai, India (**BNHS**); and the Department of Molecular Biology and Biotechnology, University of Peradeniya, Sri Lanka (**DZ**).

### Comparative material

***Amblypharyngodonmelettinus*** (all from India): MNHN 3812, *Leuciscusmelettina*, 5, syntypes, 60.7–65.7 mm SL, India, Bombay; WHT 7419, 6, 57.9–75.2 mm SL, Kerala, Kumarakom, Kottayam; WHT 7420, 6, 59.9–74.5 mm SL, Kerala, Kumarakom, Kottayam; WHT 30329–31, 3, 45.8–51.4 mm SL, Kerala, Nedumudi, Alappuzha; WHT 30787, 4, 72.5–77.3 mm SL, Kerala, Changanasserry to Alappuzha. Cleared and stained: WHT 11072, 57.6 mm SL, Kerala and WHT 11079, 49.6 mm SL, Kerala.

**A.cf.mola**: BNHS 560–61, 2, 44.5–56.8 mm SL, India, Maharashtra, Vaitarna River, Tilase.

***A.mola***: WHT30316, 5, 43.2–47.5 mm SL, India, 24 Parganas, West Bengal.

***A.microlepis***: AMS B7593, 62.5 mm SL, India, Orissa.

***A.atkinsonii***: AMS B7865, *Molaatkinsonii*, syntype, 79.4 mm SL, Myanmar, Prome [Pyay].

### Morphometric analysis

A Principal Component Analysis (PCA) in a covariance matrix was carried out to identify variables that best discriminate among the species. In view of one of us (HS) having measured the recent material and another (RP) having measured the historical material (the types of *Leuciscusmelettinus* and *Amblypharyngodongrandisquamis*), and in order to account for the deforming of specimens through long-term storage in preservative, the respective datasets were analysed separately. Prior to the PCA, all measurements were size-corrected by using the equation


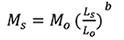


where *M*_s_ is the size corrected measurement, *M*_o_ is the measured character, *L*_s_ is the overall (arithmetic) mean standard length for all individuals from all populations of all putative species, and *L*_o_ is the standard length of the specimen, while *b* is calculated for each character from the observed data by using the allometric-growth equation *M* = *aL^b^* in which *b* is the gradient of regression of log *M*_o_ on log *L*_o_ (Elliott et al. 1995). The software PAST (Hammer et al. 2001) was used to carry out the PCA.

### Molecular analysis

The protocols for DNA extraction, PCR amplification and PCR product purification for the mitochondrial cytochrome oxidase subunit 1 (COI) and 16s rRNA subunit (16s) follow Sudasinghe et al. (2018a), while those for the mitochondrial cytochrome b (cytb) gene follow Sudasinghe et al. (2018b).

Sequences were checked and assembled in ChromasPro v1.34 (Technelysium Pty Ltd) and contig sequences of the two strands were constructed using MEGA v.7.0 (Kumar et al. 2016). The 16s, COI, and cytb contig datasets were prepared and aligned separately using ClustalW in MEGA v. 7.0 (Kumar et al. 2016) and verified manually. Protein-coding genes (COI and cytb) were translated and checked for internal stop codons or frameshift mutations. Details of the specimens used in the molecular analysis, together with additional GenBank sequences used, are given in Table 1. The uncorrected pairwise genetic distances for species of *Amblypharyngodon* for the three genes were calculated using MEGA. For each independent dataset, 16s (497 bp) and COI (654 bp), and a combined dataset of 16s+COI (1151 bp), a Bayesian phylogenetic inference was carried out using MrBayes v3.2 (Ronquist et al. 2012). A phylogenetic analysis for cytb was not carried out due to the paucity of comparative sequences in GenBank. For the Bayesian phylogenetic inference , the best-fitting nucleotide substitution model for each gene partition (16s and COI) was selected using jModelTest v.2.1.6 (Guindon and Gascuel 2003, Darriba et al. 2012) under the Bayesian information criterion (BIC). Four Metropolis coupled Markov chain Monte Carlo (MCMCMC) chains were run for 1 million generations for each analysis with a sample frequency of 100. The first 0.1% of generations was discarded as burn-in, after determining with Tracer v1.6 (Rambaut et al. 2014). The posterior probabilities (PP) of the clades (Huelsenbeck et al. 2001) were computed by using the frequency of the remaining clades in trees that were sampled every one hundred generations. Figtree v.1.4.3 (http://tree.bio.ed.ac.uk/software/figtree) was used to visualise the trees obtained from the Bayesian analyses.

**Table 1.** Species included in the phylogenetic analysis, with their localities, voucher references, and GenBank accession numbers.

Species	Voucher	Location	GPS coordinates	Source	16s	COI	cytb
* Amblypharyngodon grandisquamis *	DZ 1504	Sri Lanka: Mahaweli River basin, Ulhitiya	07.38N 81.09E	This study	MH884614	MH884634	MH884638
	DZ 3175	Sri Lanka: Mahaweli River basin, Polonnaruwa	07.87N 80.96E	This study	MH884615	MH884633	MH884639
	DZ 3176	Sri Lanka: Mahaweli River basin, Polonnaruwa	07.87N 80.96E	This study	MH884616	MH884632	MH884643
	DZ 3434	Sri Lanka: Mahaweli River basin, Naula, Matale	07.52N 80.64E	This study	MH884617	MH884628	NA
	WHT 81	Sri Lanka: Mahaweli River basin, Elahera	NA	This study	MH884618	NA	MH884645
	WHT 92	Sri Lanka: Mahaweli River basin, Minneriya	NA	This study	MH884619	NA	MH884646
	DZ 3376	Sri Lanka: Walawe River basin, Samanala reservoir	06.69N 80.78E	This study	MH884620	MH884630	MH884640
	DZ 3377	Sri Lanka: Walawe River basin, Samanala reservoir	06.69N 80.78E	This study	MH884621	MH884629	MH884641
	DZ 3292	Sri Lanka: Kalu River basin, Remuna, Horana	06.69N 80.07E	This study	MH884622	MH884631	MH884642
	DZ 3854	Sri Lanka: Attanagalu Oya basin, Yakkala	07.07N 80.07E	This study	MH884623	MH884637	NA
	DZ 3855	Sri Lanka: Attanagalu Oya basin, Yakkala	07.07N 80.07E	This study	MH884624	MH884636	NA
	DZ 3856	Sri Lanka: Attanagalu Oya basin, Yakkala	07.07N 80.07E	This study	MH884625	MH884635	NA
	WHT 38	Sri Lanka: Menik River basin	NA	This study	MH884626	NA	MH884644
	WHT 101	Sri Lanka: Kala Oya basin, Nocchiyagama	NA	This study	MH884627	NA	MH884647
* Amblypharyngodon melettinus *	Am2	India: Kerala	NA	GenBank	FJ751268	NA	NA
	Am1	India: Kerala	NA	GenBank	FJ751267	FJ751272	NA
	ACI	India: Kerala	NA	GenBank	FJ751266	FJ751271	NA
	A2	India: Kerala	NA	GenBank	FJ751265	FJ751270	NA
	A1	India: Kerala	NA	GenBank	FJ751264	FJ751269	NA
* Amblypharyngodon mola *	NBFGRMU AM8004L	India	23.32N 91.28E	GenBank	KT878048	NA	NA
	NBFGRMU AM8004K	India	23.32N 91.28E	GenBank	KT878047	NA	NA
	NBFGRMU AM8004H	India	23.32N 91.28E	GenBank	KT878046	NA	NA
	NBFGRMU 8004S	India	24.39N 93.58E	GenBank	KT878045	NA	NA
	NBFGRMU 8004M	India	24.30N 93.46E	GenBank	KT878044	KT896674	NA
	PUMNH 28/2013	India	NA	GenBank	NA	KX266827	NA
	8004Q	India	24.34N 93.46E	GenBank	NA	KX245049	NA
	8004P	India	24.34N 93.46E	GenBank	NA	KX245048	NA
	8004o	India	24.34N 93.46E	GenBank	NA	KX245047	NA
	8004N	India	24.34N 93.46E	GenBank	NA	KX245046	NA
	DUZM031	Bangladesh: Tanguar Haor, Sunamganj	25.08N 91.33E	GenBank	NA	KT364774	NA
	AM-1005	India	25.76N 89.95E	GenBank	NA	KJ936819	NA
	AM-1004	India	25.76N 89.95E	GenBank	NA	KJ936818	NA
	AM-1003	India	25.76N 89.95E	GenBank	NA	KJ936817	NA
	AM-1002	India	25.76N 89.95E	GenBank	NA	KJ936816	NA
* Amblypharyngodon mola *	AM-1001	India	25.76N 89.95E	GenBank	NA	KJ936815	NA
	AM-2005	India	24.29N 92.63E	GenBank	NA	KJ936763	NA
	AM-2004	India	24.29N 92.63E	GenBank	NA	KJ936762	NA
	AM-2003	India	24.29N 92.63E	GenBank	NA	KJ936761	NA
	AM-2002	India	24.29N 92.63E	GenBank	NA	KJ936760	NA
	AM-2001	India	24.29N 92.63E	GenBank	NA	KJ936759	NA
	NF770	India: Gujarat, Bharuch, Estuary, Bharuch	21.68N 73.45E	GenBank	NA	JX983212	NA
	NF771	India: Gujarat, Bharuch, Estuary, Bharuch	21.68N 73.45E	GenBank	NA	JX983211	NA
	PRUM01	India: Maharashtra, Vidarbha, Umarkhed Painganga	19.59N 77.68E	GenBank	NA	JX260822	NA
	GF675	India: Maharashtra, Marathwada, Kaigaon	19.62N 75.01E	GenBank	NA	JX260821	NA
	BESH02	India: Maharashtra, Shahagad River Pool Cad	19.36N 75.71E	GenBank	NA	JX260820	NA
	PSSH02	India: Maharashtra, Shahagad River Pool Cad	19.36N 75.71E	GenBank	NA	JX260819	NA
	GF679	India: Maharashtra, Marathwada, Kaigaon	19.62N 75.01E	GenBank	NA	JX260818	NA
	GDK025-11	India: Maharashtra, Shahagad River Pool Cad	19.36N 75.71E	GenBank	NA	JX260817	NA
	DOF16	India	24.46N 93.01E	GenBank	NA	JN815278	NA
	DOF15	India	24.46N 93.01E	GenBank	NA	JN815277	NA
	CTOL01907	NA	NA	GenBank	NA	HM224137	HM224256
	AM2	India	NA	GenBank	NA	NA	KX389702
	AM1	India	NA	GenBank	NA	NA	KX389701
* Amblypharyngodon chulabhornae *	NA	NA	NA	GenBank	AP012114	AP012114	AP012114
	NA	NA	NA	GenBank	U21380	NA	NA
	CTOL01544	NA	NA	GenBank	NA	NA	HM224255
* Barilius vagra *	CTOL03301	NA	NA	GenBank	NA	HM224140	HM224259
* Barilius gatensis *	4BG	India	NA	GenBank	NA	KF853168	NA
	6BG	India	NA	GenBank	KF853144	NA	NA
	5BG	India	NA	GenBank	KF853143	NA	NA
* Salmostoma phulo *	CTOL03316	NA	NA	GenBank	NA	HM224248	HM224379
* Salmostoma bacaila *	WL-F38	India	NA	GenBank	NA	EU417789	NA
	NBFGRMU 8020G	India	24.42N 93.04E	GenBank	KT878279	NA	NA
	NBFGRMU 8020F	India	24.42N 93.04E	GenBank	KT878278	NA	NA

Haplotype network reconstruction for the 16s, COI, and cytb genes of the various populations of *Amblypharyngodon* was inferred by TCS network (Clement et al. 2002) in PopArt (Leigh and Bryant 2015). DNAsp v.6 (Rozas et al. 2017) was used to compute the nucleotide diversity (π), haplotype diversity and neutrality tests: Tajima’s D (Tajima 1989) and Fu and Li’s F (Fu and Li 1993).

## Results

### Molecular analysis

**Figure 1.**
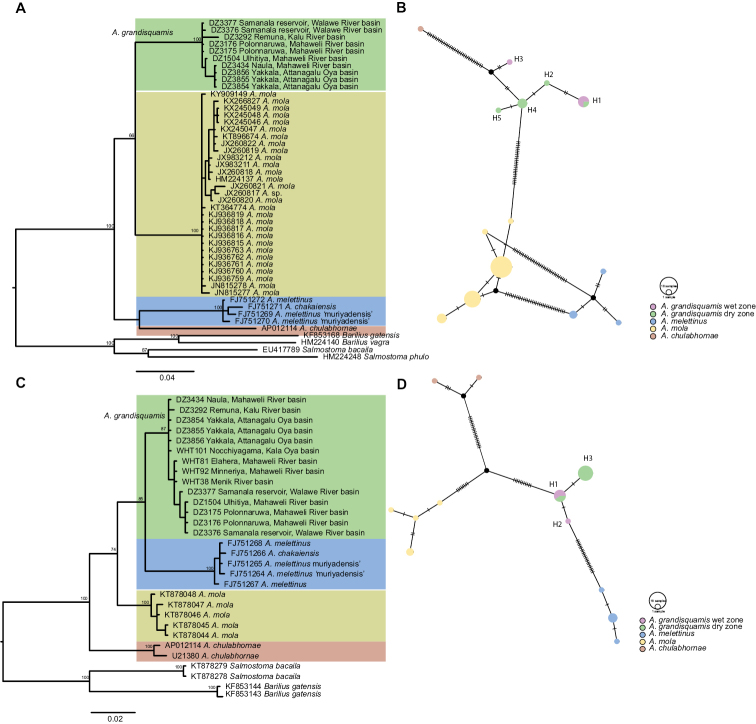
**A, C** Phylogram based on Bayesian phylogenetic inference of **A** the COI and **C**16s dataset for species of *Amblypharyngodon*. Numbers above nodes represent Bayesian Posterior Probabilities. The scale bar represents the number of changes per site **B, D** TCS haplotype network for species of *Amblypharyngodon* based on the analysis of **B** a 654-bp fragment of the COI gene and **D** a 497-bp fragment of the 16s gene. The sizes of the circles are proportional to the number of individuals sharing a given haplotype. The number of mutational steps is indicated by hatch marks. The black circles represent hypothetical nodes.

The HKY+G model was chosen as the best-fit nucleotide-substitution model under BIC in the jModelTest for the COI dataset. In the Bayesian phylogenetic inference for the COI dataset, *A.mola* was recovered as the sister species of *A.grandisquamis*, though weakly supported (PP = 66, Figure 1A). The GenBank sequence JX260817*Amblypharyngodon* sp nests within the rest of the sequences of *A.mola*.

The SYM+G model was chosen as the best-fit nucleotide-substitution model under BIC in the jModelTest for the Bayesian phylogenetic inference of the 16s dataset. Here, *Amblypharyngodonmelettinus* was recovered as the sister species of *A.grandisquamis* (PP = 85, Figure 1C). The GenBank sequences FJ751266 as *A.chakaiensis*, FJ751264–65 as *A.melettinus* ‘muriyadensis’ nest with the other sequences of *A.melettinus* (FJ751267–68) available in GenBank.

In the combined 16s+COI (1151 bp) Bayesian phylogenetic inference, *A.mola* was recovered as the sister species of *A.grandisquamis* (PP = 89) while *A.melettinus* was recovered as the sister species of [*A.grandisquamis* + *A.mola*] (PP = 100).

The uncorrected pairwise genetic distances obtained for the 16s, COI, and cytb genes for the species of *Amblypharyngodon* is given in Table 2. *Amblypharyngodongrandisquamis* differs genetically from *A.melettinus*, *A.mola*, and *A.chulabhornae* by uncorrected pairwise distances of more than 4% for the 16s gene fragment, while the intraspecific divergence within *A.grandisquamis* for the same gene is only 0.0–0.4%. For COI, *A.grandisquamis* differs from *A.melettinus*, *A.mola*, and *A.chulabhornae* by uncorrected pairwise genetic distances of more than 9%, 6%, and 10%, respectively, while the intraspecific genetic divergence within *A.grandisquamis* for the same gene is only 0.0–1.9%. With respect to cytb, *A.grandisquamis* differs from *A.mola* and *A.chulabhornae* by uncorrected pairwise genetic distances of more than 7% and 14%, respectively, while the intraspecific genetic divergence within *A.grandisquamis* for the same gene is 0.0–4.2% (there are no cytb sequences available for *A.melettinus*). DZ 3292 from Remuna, Kalu basin (wet zone) differs from the other Sri Lankan samples by 3.2–4.2%, while WHT 101 from Nochiyagama, Kala Oya basin (dry zone) differs from them by 1.8–2.5%.

**Table 2.** Intraspecific and interspecific percentage uncorrected pairwise genetic distances, for the 16s, COI, and cytb genes, between species of *Amblypharyngodon*.

	*** A. grandisquamis ***			*** A. melettinus ***			*** A. mola ***			*** A. chulabhornae ***		
%	**16s**	**COI**	**cytb**	**16s**	**COI**	**cytb**	**16s**	**COI**	**cytb**	**16s**	**COI**	**cytb**
* A. grandisquamis *	0.0–0.4	0.0–1.9	0.0–4.2									
* A. melettinus *	4.4–5.2	9.9–11.0	NA	0.0–0.4	0.8–1.1	NA						
* A. mola *	4.0–4.6	6.6–8.3	7.0–9.2	5.6–6.3	9.6–10.7	NA	0.0–0.6	0.0–0.8	0.0–0.4			
* A. chulabhornae *	6.3–6.9	10.2–10.5	14.8–15.1	7.3–7.9	10.7–11.6	NA	5.9–6.7	10.7–11.0	14.4–14.8	0.8	NA	0.0

In the COI haplotype network, the dry zone and wet zone samples of *A.grandisquamis* form a shared a haplotype (H1) (Figure 1B). A single unique haplotype (H3) occurs in the wet zone, while three unique haplotypes are recorded from the dry zone (H2, H4, H5) (Figure 1B). Populations of *A.grandisquamis* (ten sequences) included 12 segregating sites and five parsimony-informative sites. The nucleotide diversity and haplotype diversity for *A.grandisquamis* were 0.0073 and 0.800, respectively. Tajima’s D test and Fu and Li’s F* test statistic were both negative (-0.16285, -0.65621) but not significant (p > 0.05, p > 0.02).

Only three haplotypes occur in the 16s haplotype of *A.grandisquamis* (Figure 1D). These include a haplotype (H1) shared between populations of the dry and wet zones, together with a unique dry zone (H3) and unique wet zone (H2) haplotype. Populations of *A.grandisquamis* (14 sequences) included two segregating sites and a single parsimony-informative site. The nucleotide diversity and haplotype diversity for *A.grandisquamis* were 0.00139 and 0.582, respectively. Tajima’s D test was positive (0.17874) and Fu and Li’s F* test statistic was negative (-0.32441), but neither was significant (p > 0.05, p > 0.02).

In the cytb haplotype network for *A.grandisquamis*, the only representative of the wet zone population (DZ 3292) in our molecular analysis formed a unique haplotype, while the dry zone samples formed four unique haplotypes. Populations of *A.grandisquamis* (ten sequences) included 22 segregating sites and three parsimony-informative sites. The nucleotide diversity and haplotype diversity for *A.grandisquamis* were 0.0101 and 0.667, respectively. Although Tajima’s D test was positive (1.7016) and Fu and Li’s F* test statistic was negative (-2.0688), neither was significant (p > 0.05, p > 0.02, respectively).

### Statistical analysis

**Figure 2.**
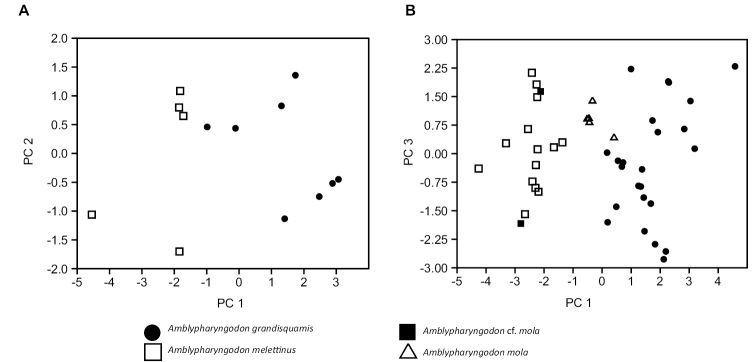
Plot of scores from the principal component analysis of size-corrected measurements of **A** paratypes of *Amblypharyngodongrandisquamis* (SU 22868) and syntypes of *A.melettinus* (MNHN 3812), and **B** recent material examined of *A.grandisquamis*, *A.melettinus*, *A.mola*, and A.cf.mola.

The morphometric PCA of the syntypes of *A.melettinus* (MNHN 3812) and the paratypes of *A.grandisquamis* (SU 22868) clearly separate the two species into two distinct clusters, with body depth and caudal-peduncle length explaining most of the variation (Figure 2A, Table 3). Similarly, the PCA of the recent material separate *A.grandisquamis*, *A.melettinus*, and *A.mola* in morphological space (Figure 2B, Table 4), with only a slight overlap between *A.grandisquamis* and *A.mola*. The identity of the two specimens identified as A.cf.mola (BNHS 560–61) is doubtful (see Discussion). Most of the variation on PC1 is explained by pre-anal length and body depth, while the variation on PC3 is explained mostly by the dorsal-fin height.

**Table 3.** Component loadings in the principal component analysis of the size-adjusted morphometric measurements of the paratypes of *Amblypharyngodongrandisquamis* (SU 22868) and syntypes of *A.melettinus* (MNHN 3812).

	**Component 1**	**Component 2**	**Component 3**
Eigenvalue	5.4435	0.9590	0.6933
Variance explained	73.725	12.989	9.3901
Body depth	0.8028	0.5239	-0.1882
Caudal peduncle length	0.5863	-0.7560	0.1355
Caudal peduncle depth	-0.0145	-0.0398	-0.4534
Head length	0.0500	0.1730	0.8129
Snout length	-0.0563	0.1567	-0.1712
Eye diameter	-0.0286	-0.0125	0.0303
Inter-orbital width	0.0698	0.3125	0.2225

**Table 4.** Component loadings in the principal component analysis of the size-adjusted morphometric measurements of the recent material of *Amblypharyngodon* examined in the study.

	**Component 1**	**Component 2**	**Component 3**
Eigenvalue	4.4466	3.2916	1.8519
Variance explained	30.144	22.314	12.554
Predorsal length	0.0175	0.3200	-0.0137
Postdorsal length	0.2933	-0.2495	-0.1115
Preanal length	-0.4055	0.2789	0.2023
Prepelvic length	-0.2027	0.2117	0.1323
Caudal peduncle length	0.3304	0.0390	-0.3040
Caudal peduncle depth	0.1731	-0.0041	0.0432
Body depth	0.5663	-0.0706	0.1794
Dorsal-fin height	0.0970	-0.2158	0.5209
Dorsal-fin base length	0.0238	-0.1490	0.1537
Anal-fin height	0.0730	-0.0733	0.3317
Anal-fin base length	0.0095	-0.1455	0.1407
Pelvic-fin height	0.0025	0.0514	0.3114
Pelvic-fin base length	0.0096	0.0160	0.0913
Pectoral-fin length	0.0484	-0.2036	0.3004
Pelvic-anal distance	-0.1957	0.0167	0.1557
Head length	0.2971	0.6690	0.0630
Head depth	0.2368	0.3131	0.3509
Snout length	0.0013	0.0617	0.0177
Eye diameter	-0.0671	0.0275	0.0929
Post-orbital head length	0.1730	0.1212	-0.01411
Inter-orbital width	0.1131	0.0311	-0.0267
Inter-narial width	-0.0175	0.0100	0.0304

#### Amblypharyngodon grandisquamis

Taxon classificationAnimaliaCypriniformesCyprinidae

Jordan & Starks, 1917

Figure 4

Amblypharyngodon melettinus , Non-Valenciennes, 1844 (from Sri Lanka): Günther 1868: 202; Day 1878: 555; Day 1889: 292; Duncker 1912: 265; Deraniyagala 1952: 45; Senanayake 1980: 167; Pethiyagoda 1991: 25; Pethiyagoda 2006; MOE 2012; De Silva et al. 2015.

##### Material examined (all from Sri Lanka).

SU 22868, 8 **paratypes**, 56.4–65.4 mm SL, river at Colombo (presumably the Kelani, which is the only river in the vicinity of the city); WHT 30275, 6, 65.0–76.4 mm SL, Bolgoda River basin, Bellanwila-Attidiya; WHT 64, 3, 45.4–47.5 mm SL, Kalu River basin, Ingiriya, Dombagaskanda; WHT 22, 2, 37.2–38.7 mm SL, Kalu River basin, Walandure, Kuruwita; WHT 225, 2, 30.2–34.3 mm SL, Kalu River basin, Ekneligoda, Kuruwita; WHT 30790, 3, 49.3–52.8 mm SL, Kelani River basin, Deraniyagala; WHT 32, 3, 49.6–60.1 mm SL, Attanagalu Oya basin, Weliweriya; WHT 30260, 58.2 mm SL, Nilwala River basin, Godapitiya, Akuressa; WHT 30167, 3, 54.2–74.7 mm SL, Malwathu Oya basin, Anuradhapura; DZ 3193, 7, 57.2–74.0 mm SL, Mahaweli River basin, Polonnaruwa; DZ 3564, 2, 57.2–62.3 mm SL, Mahaweli River basin, Badulu Oya, Kandeketiya; WHT 1694, 3, 42.7–45.1 mm SL, Mahaweli River basin, Wasgamuwa; WHT 45, 4, 54.6–71.9 mm SL, Mi Oya basin, Puttlam; WHT 1810, 4, 40.8–52.4 mm SL, Kirindi Oya basin, Weerawila; WHT 1851, 41.2 mm SL, Gal Oya basin, Rathugala near Bibile. **Cleared and stained**: WHT 11075, 64.2 mm SL, Bolgoda River basin, Bellanwila-Attidiya; WHT 11046, 54.2 mm SL, Mi Oya basin, Puttlam; WHT 11081, 45.1 mm SL, Mi Oya basin, Puttlam; WHT 11052, 45.9 mm SL, Kelani River basin, Deraniyagala; WHT 11070, 40.0 mm SL, Kelani River basin, Deraniyagala. **Other material** (identified for distribution data, but not measured): WHT 30789, Mahaweli River basin, Wasgamuwa; DZ 3434, Mahaweli River basin, Naula; DZ 1504, Mahaweli River basin, Ulhitiya; WHT 30263, Deduru Oya basin, Wewagama, Kuliyapitiya; WHT 30679, Deduru Oya basin; WHT 2158, Kirindi Oya basin, Tissamaharama; DZ 4423, Kirindi Oya basin, Lunugamwehera; WHT 30723, Heda Oya, Lahugala; WHT 30310, Kala Oya basin, Eluwankulam; WHT 30167, Malwathu Oya basin, Anuradhapura; DZ 4042, Attanagalu Oya basin, Yakkala; DZ 3577, Mi Oya basin, Galgamuwa; DZ 3292, Kalu River basin, Remuna.

##### Diagnosis.

**Figure 3.**
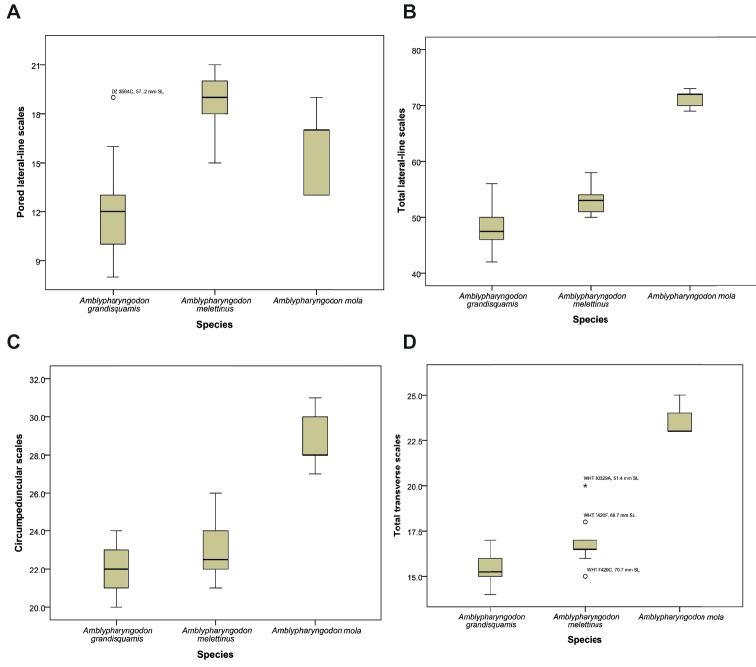
Boxplots representing the distribution of **A** pored lateral-line scales **B** lateral series scales **C** circumpeduncular scales and **D** total transverse scales in recent material of *A.grandisquamis*, *A.melettinus*, and *A.mola*.

*Amblypharyngodongrandisquamis* is distinguished from *A.melettinus* by having a deeper body (26.9–31.2% SL in *A.grandisquamis*, vs. 22.9–26.3 in *A.melettinus*); fewer pored lateral-line scales (8–16 (25), 19 (1), vs. 15(1), 17–21 (13) in *A.melettinus*: Figure 3A); more caudal vertebrae (18–19 (5), vs. 17 (2) in *A.melettinus*); 2–4 minute foramina (absent in *A.melettinus*) in addition to a large foramen on the base of the lateral arm of the fifth ceratobranchial; and absence of a minute foramen at the base of the medial arm of the fifth ceratobranchial (present in *A.melettinus*). It differs from *A.microlepis* by having fewer scales in the lateral series (42–56 vs. 55–65 in *A.microlepis*) and a greater body depth (26.9–31.2% SL vs. 24.3–26.3 in *A.microlepis*); from *A.mola* by a lesser dorsal-fin height (21.1–27.6% SL vs. 27.8–29.2 in *A.mola*), shorter eye diameter (22.7–30.5% HL vs. 31.3–36.6 in *A.mola*), fewer scales in the lateral series (42–56 vs. 69–73 in *A.mola*: Figure 3B), fewer circumpeduncular scales (20–24 vs. 27–31 in *A.mola*: Figure 3C), and fewer scale rows between the origins of the dorsal and pelvic fins (14–17 vs. 23–25 in *A.mola*: Figure 3D); from *A.chulabhornae* by having more pored lateral-line scales (8–16 (25) or 19 (1) vs. 6–7 in *A.chulabhornae*) and more vertebrae (33–34 (5) vs. 31–32 in *A.chulabhornae*); and from *A.atkinsonii* by having fewer scales in the lateral series (42–56 vs. 55–61 in *A.atkinsonii*), a lesser body depth (26.9–31.2% SL, vs. 40.5 in *A.atkinsonii*), and fewer scale rows between the origins of the dorsal and pelvic fins (14–17 vs. 21 in *A.atkinsonii*).

##### Description.

**Figure 4.**
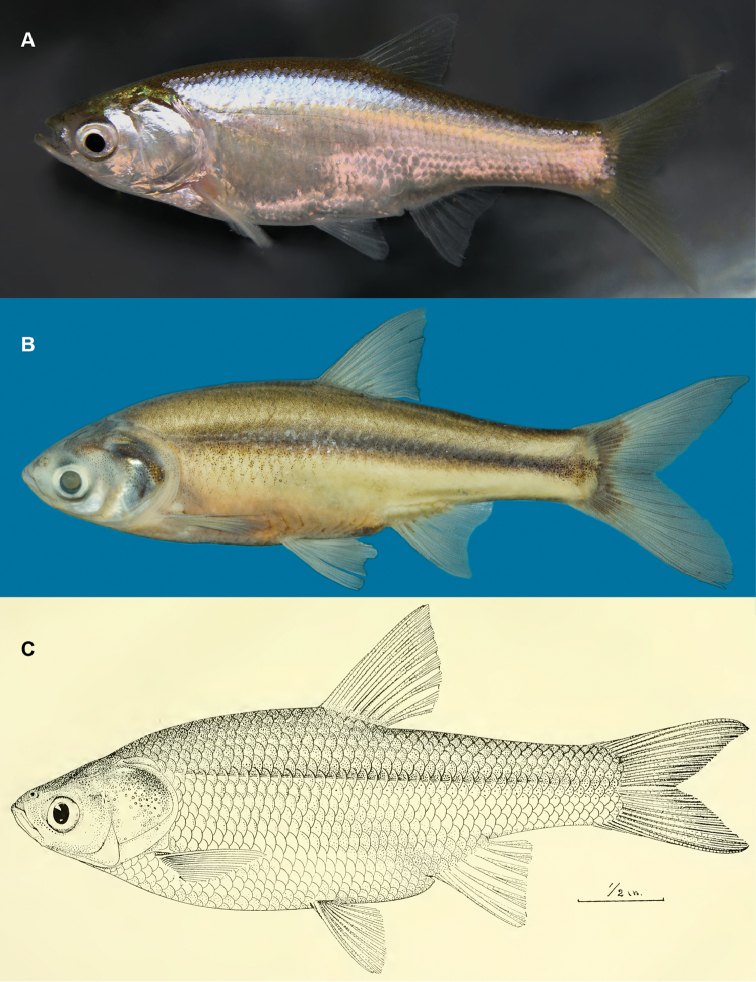
*Amblypharyngodongrandisquamis*, **A** in aquarium (~70 mm SL, not collected), Sri Lanka, Ulhitiya, Mahaweli River basin **B** in preservation (DZ 3193A, 74.0 mm SL), Sri Lanka, Polonnaruwa, Mahaweli River basin **C** illustration of the holotype of *A.grandisquamis* by Jordan and Starks (1917: pl. XLIV, FMNH 58964).

For general appearance, see Figure 4; morphometric data are provided in Table 5. Head and body oblong, slightly compressed. Head wider than body. Body depth greatest at dorsal-fin origin. Snout short, subequal to eye diameter, dorsally rounded, laterally subtriangular. Mouth terminal; symphysial knob present, small, elongated, fitting into shallow groove on inner margin of upper jaw with mouth closed.

**Table 5.** Morphometric data for *Amblypharyngodongrandisquamis* (SU 22868, WHT 30275, WHT 64, WHT 30790, WHT 32, WHT 30260, WHT 30167, WHT 45, DZ 3193, DZ 3564), *A.melettinus* (MNHN 3812, WHT 7419, WHT 7420, WHT 30329, WHT 30787), and *A.mola* (WHT 30316).

	* A. grandisquamis *								* A. melettinus *								*A.mola* (n = 5)			
	paratypes of *A.grandisquamis* (n = 8)				recently collected material of *A.grandisquamis* (n = 23)				syntypes of *A.melettinus* (n = 5)				recently collected material of *A.melettinus* (n = 14)							
	min	max	mean	s.d.	min	max	mean	s.d.	min	max	mean	s.d.	min	max	mean	s.d.	min	max	mean	s.d.
Standard length (mm)	56.4	65.4	–	–	45.4	76.4	–	–	60.7	65.7	–	–	51.4	77.3	–	–	43.2	47.5	–	–
**As percentage of standard length**																				
Total length	–	–	–	–	125	128	127	0.9	–	–	–	–	126	131	128	1.4	126	132	130	2.6
Predorsal length	–	–	–	–	52.8	57.7	55.2	1.3	–	–	–	–	53.8	56.7	55.2	0.8	51.5	56.3	54.7	1.9
Postdorsal length	–	–	–	–	47.4	53.1	50.4	1.5	–	–	–	–	46.9	50.0	48.6	0.9	48.9	52.6	50.3	1.5
Preanal length	–	–	–	–	64.3	69.4	67.4	1.3	–	–	–	–	68.9	72.6	70.6	1.3	67.8	69.7	68.5	0.8
Prepelvic length	–	–	–	–	47.6	52.3	49.7	1.2	–	–	–	–	48.8	53.1	50.9	1.4	48.6	53.9	50.3	2.1
Caudal peduncle length	19.9	24.2	22.7	2.1	21.1	25.7	23.9	1.1	17.8	21.1	18.9	1.3	20.4	23.8	21.7	0.8	19.4	22.1	21.3	1.1
Caudal peduncle depth	10.6	13.3	12.0	0.9	11.4	13.9	12.5	0.6	12.2	13.2	12.7	0.4	10.3	12.4	11.3	0.7	10.7	11.8	11.2	0.5
Body depth	29.3	33.6	31.8	1.6	26.9	31.2	29.0	1.2	23.2	28.8	26.6	2.3	22.9	26.3	24.7	1.0	28.0	29.7	28.7	0.7
Dorsal–fin height	–	–	–	–	21.1	27.6	24.5	2.0	–	–	–	–	21.9	26.1	23.8	1.3	27.8	29.2	28.2	0.6
Dorsal–fin base length	–	–	–	–	10.6	13.6	12.0	0.8	–	–	–	–	10.0	13.5	11.7	1.0	12.0	14.1	13.2	0.8
Anal–fin height	–	–	–	–	14.6	19.8	17.1	1.5	–	–	–	–	15.5	19.4	16.8	1.0	17.3	21.3	18.8	1.5
Anal–fin base length	–	–	–	–	10.1	13.3	11.7	0.8	–	–	–	–	10.0	12.6	11.6	0.9	13.0	15.1	14.1	0.9
Pelvic–fin height	–	–	–	–	14.7	19.4	16.8	1.2	–	–	–	–	15.8	18.6	16.9	0.9	17.5	19.6	18.7	0.8
Pelvic–fin base length	–	–	–	–	4.2	5.7	4.9	0.4	–	–	–	–	3.7	6.8	5.0	0.9	3.7	5.3	4.6	0.7
Pectoral fin height	–	–	–	–	15.6	21.5	18.6	1.5	–	–	–	–	17.2	19.9	18.2	0.7	20.0	22.3	21.4	1.0
Pelvic–anal distance	–	–	–	–	17.5	20.9	19.7	0.9	–	–	–	–	19.7	23.9	21.4	0.9	18.7	22.0	20.0	1.4
Head length	26.9	30.9	28.7	1.2	24.6	39.7	28.5	2.7	25.8	28.9	27.7	1.3	26.1	29.0	27.4	1.0	25.9	29.4	27.7	1.4
Head depth	–	–	–	–	18.2	31.5	21.1	2.4	–	–	–	–	18.5	20.3	19.6	0.6	20.4	22.5	21.4	0.8
**As percentage of head length**																				
Snout length	20.1	26.0	23.5	1.8	19.3	29.2	24.3	2.6	25.4	29.8	27.0	1.8	23.4	28.6	25.8	1.6	23.7	27.5	24.8	1.5
Eye diameter	24.1	26.9	26.2	1.2	22.7	30.5	27.0	2.6	26.8	30.4	28.2	1.5	25.2	33.5	28.8	2.3	31.3	36.6	33.7	2.5
Post–orbital head length	–	–	–	–	39.6	57.5	52.9	3.5	–	–	–	–	45.6	53.6	50.3	2.5	48.9	52.8	50.2	1.5
Inter–orbital width	31.9	36.6	34.7	1.6	24.4	42.3	36.6	3.5	28.3	38.5	34.9	3.9	31.8	38.0	34.8	1.5	32.8	37.1	34.9	1.7
Inter–narial width	–	–	–	–	14.7	28.6	21.8	2.7	–	–	–	–	19.5	25.3	22.1	1.9	22.1	24.4	23.3	1.2

Lateral line incomplete, with 8 (1), 9 (2), 10 (4), 11 (4), 12 (6), 13 (5), 15 (1), 16 (2) or 19 (1) pored scales, 42 (1), 44 (1), 45 (4), 46 (3), 47 (4), 48 (4), 49 (2), 50 (1), 51 (1), 52 (2), 53 (2) or 56 (1) scales in lateral series plus 2–4 scales on base of caudal fin. Predorsal scales 27 (2), 28 (3), 29 (2), 30 (2) or 32 (1). Prepelvic scales 25 (1), 26 (1), 27 (1), 28 (1), 29 (1), 30 (2), 31 (1), 33 (1) or 34 (1). Lateral scale rows between origins of dorsal and pelvic fins ½8+1+5½ (1), ½8+1+6 (1), ½9+1+4½ (2), ½9+1+5½ (8), ½9+1+6 (1), ½9+1+6½ (2), ½10+1+4½ (1), ½10+1+5 (1), ½10+1+5½ (4), or ½10+1+6½ (5). Circumpeduncular scales 20 (2), 21 (7), 22 (10), 23 (5) or 24 (2).

Dorsal fin with two unbranched and seven branched rays, its origin just posterior to vertical through pelvic-fin origin, its distal margin straight. Anal fin with three unbranched and five branched rays, its origin slightly posterior to vertical through origin of dorsal fin, its distal margin slightly concave. Pectoral fin with a single unbranched and 11 (1), 12 (10), 13 (2), or 14 (1) branched rays, its origin anterior to posteriormost point of opercular opening, not reaching pelvic-fin origin when adpressed. Pectoral-fin axillary lobe rudimentary. Pelvic fin with one unbranched and 7 (3) or 8 (13) branched rays, its origin slightly closer to anal-fin origin than to origin of pectoral fin, its tip not reaching anal-fin origin when adpressed. Pelvic ‘axillary’ scale present. Caudal fin with 9 + 8 (14) branched rays, forked, lobes rounded distally, upper and lower lobes subequal.

**Figure 5.**
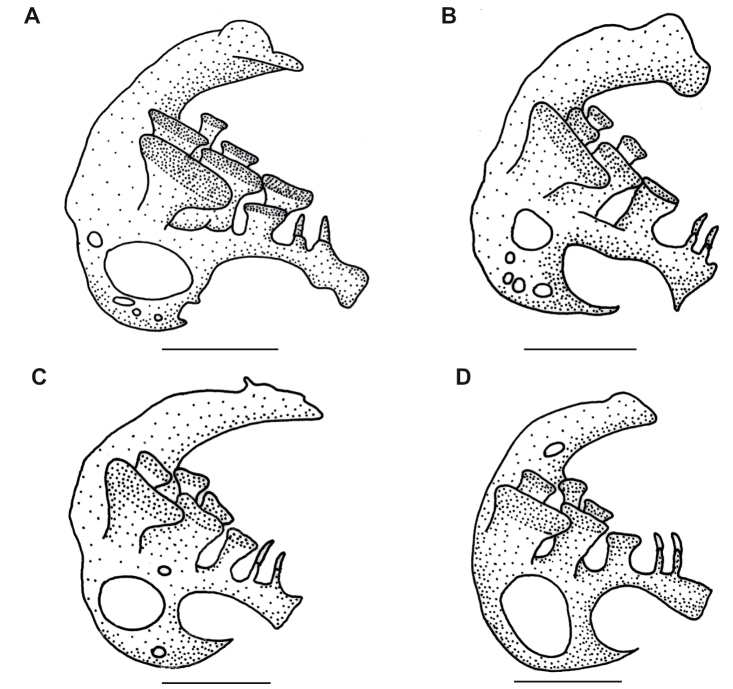
Comparison of the fifth ceratobranchial of **A–C***Amblypharyngodongrandisquamis* and **D***A.melettinus*. **A**WHT 11075, 64.2 mm SL **B**WHT 11046, 54.2 mm SL **C**WHT 11070, 40.0 mm SL **D**WHT 11072, 57.6 mm SL. Scale bar: 1 mm.

Vertebrae 15 + 18 = 33 (1), 15 + 19 = 34 (4). Pharyngeal teeth 5 + 2 + 1 (4), 5 + 3 + 1 (1) (Figure 5A–C). Fifth ceratobranchial with 2–4 minute foramina in addition to a large foramen at base of lateral arm; no foramen at base of medial arm (Figure 5A–C).

##### Coloration.

In 70% alcohol (Figure 4B), head and body silvery brown, darker dorsally, becoming lighter laterally, off-white ventrally. Head darker than body. Dusky-brown stripe 1–1½ scales wide on side of body, from immediately behind operculum, extending to caudal fin base, broader at middle, scales above it with prominent melanophores throughout, scales below it with scattered melanophores on margins, disappearing ventrally. Fins hyaline. Dorsal and caudal fins with scattered melanophores, more prominent on caudal fin.

In life (Figure 4A), head and body silvery grey to iridescent gold, lighter laterally. Scattered melanophores on side of body. A faint yellowish stripe extending from behind operculum to caudal fin base. Caudal fin yellowish, other fins mostly hyaline.

##### Habitat, distribution, and natural history.

**Figure 6.**
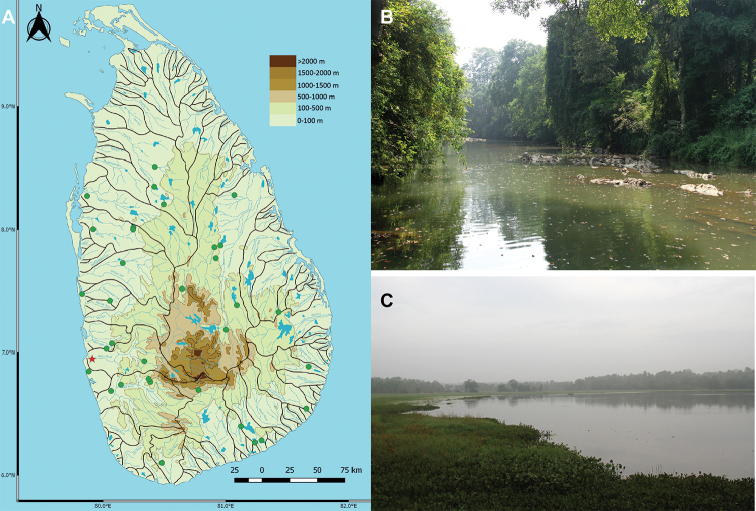
**A** Distribution of *Amblypharyngodongrandisquamis* in Sri Lanka. Present records (green) and the type locality (red); habitat of *A.grandisquamis* in **B** Badulu Oya, Kandeketiya and **C** a reservoir at Galgamuwa.

*Amblypharyngodongrandisquamis* occurs in lotic habitats such as rivers and canals as well as in lentic habitats such as reservoirs and marshes. The species is recorded primarily from the lowland floodplain of Sri Lanka, in both the dry and the wet zones of the island (annual precipitation less than, and greater than, 2,000 mm, respectively), though much more frequently encountered in the dry zone (Figure 6). The highest elevation from which we recorded *A.grandisquamis* was at ca 460 m a.s.l., in the Samanala reservoir on the Walawe River basin. It is a slow-swimming fish, usually encountered in large groups close to the surface. With the onset of the rains, adults are observed in rice paddies, probably migrating there to spawn. The relative abundance of *A.grandisquamis*, at least in the wet zone, appears to be seasonal, with more adults usually observed during the rainy season.

## Discussion

While Menon (1999) did not consider *A.mola* to occur in the peninsula of India, most other recent authors (e.g., Talwar and Jhingran 1991, Jayaram 2010), following Day (1878), recognise three species of *Amblypharyngodon* from the peninsula: *A.melettinus, A.microlepis* and *A.mola*. We therefore discuss the identity of each of these in the context of *A.grandisquamis*.

### *Amblypharyngodon melettinus*

The species we here refer to *Amblypharyngodongrandisquamis* was first reported from Sri Lanka by Günther (1868: 202) and Day (1878: 555), as *Amblypharyngodonmelettinus* (Valenciennes 1844). Although Günther (1868) gave the distribution of *A.melettinus* as “Bombay; Coast of Malabar; Ceylon [= Sri Lanka]”, it is clear from the listing of material at the end of his description that he had not seen specimens from Bombay. For his part, Day (1878) had the distribution of this species as “(Bombay, according to Cuv. and Val.) Malabar coast, and southern India, from the Neilgherries to Madras”, making it clear that he too, had not seen specimens from Bombay. As such, the conception of *A.melettinus* by these authors was based essentially on the original description of Valenciennes (1844) and specimens from southern peninsular India and Sri Lanka: neither Günther (1868) nor Day (1878) had opportunity to examine the type specimens in Paris.

**Figure 7.**
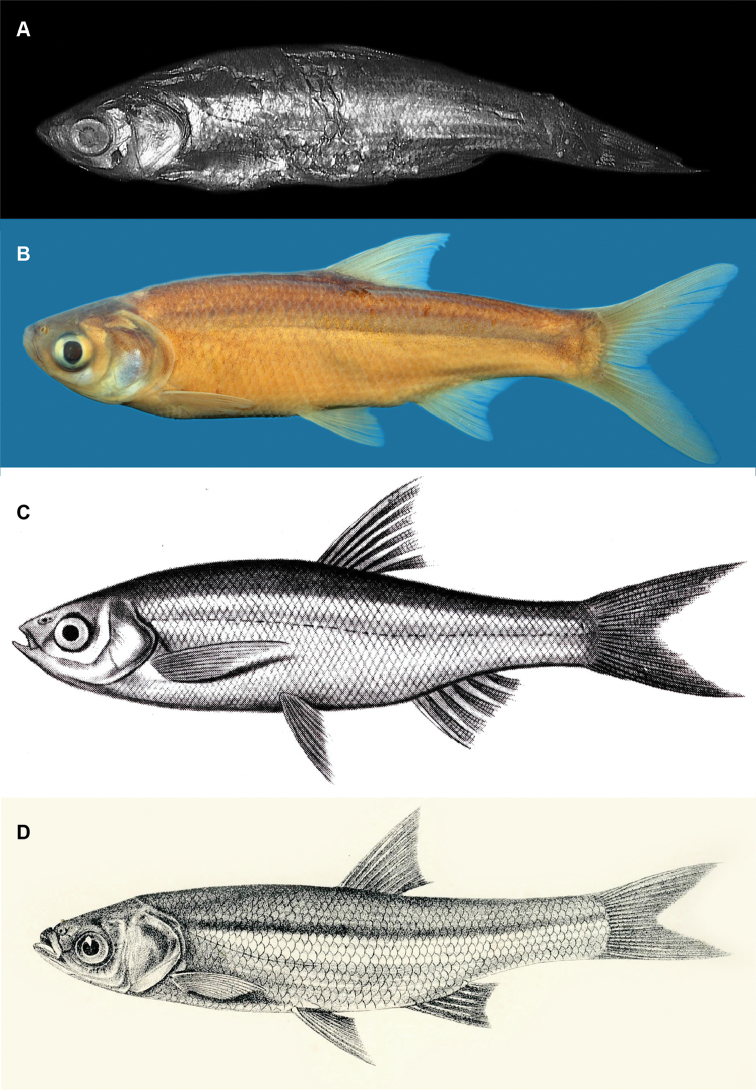
**A** syntype of *Amblypharyngodonmelettinus*, MNHN 3812, 65.7 mm SL, India, Bombay **B** specimen of *A.melettinus*, WHT 7419A, 72.2 mm SL, India, Kerala, Kumarakom, Kottayam **C** illustration of *A.melettinus* by Valenciennes (1844: pl. 501) **D** illustration of *A.melettinus* by Day (1878: pl. CXXXIV, fig. 3), laterally inverted.

The syntypes of *A.melettinus* are in quite poor condition. Jean-Jacques Dussumier probably collected these on his visit to Bombay 1827–30 (see Bauchot et al. 1990: 46). We have been unable to determine whether, when Valenciennes (1844) gave the type locality as Bombay, he meant the city of Bombay (now Mumbai) or the erstwhile Bombay Presidency. The latter, in the mid-19^th^ century, encompassed a vast territory inland of British India’s Arabian-sea coastline, extending from southern Karnataka across the Sindh Province of present-day Pakistan and on to Yemen (Collen 1909). The result of this uncertainty has been that subsequent authors (e.g., Talwar and Jhingran 1991, Menon 1999, Jayaram 2010) appear to have followed Day’s (1878) conception of *A.melettinus* as the species of *Amblypharyngodon* inhabiting the south-western region of the Indian peninsula, characterised by the lateral line being incomplete, with 15–20 pored scales, and with 50–57 scales in the lateral series and 4 scale-rows between the lateral-line row and the pelvic-fin origin. These counts are broadly consistent with the original description of *A.melettinus*, “more than fifty rows of scales along the flanks…” (Valenciennes 1844: 305) and those syntypes in which scales can reliably be counted. The latter possess 16–21 pored lateral-line scales and 48–55 scales in the lateral series, plus a further 2–3 on the caudal-fin base. While these counts overlap partly with those of *A.grandisquamis* (8–16, rarely up to 19 pored lateral-line scales, 42–56 scales in lateral series on body), the eight paratypes of *A.grandisquamis* (SU 22868) are distinguished from the five syntypes of *A.melettinus* (MNHN 3812) by their greater body depth (29.3–33.6% SL, vs. 23.2–28.8% SL), bearing in mind that evisceration and age have resulted in the latter specimens becoming somewhat compressed and hence appearing deeper-bodied (see Figure 7A). Nevertheless, the body depth in recently collected *A.grandisquamis* (N = 23) is 26.9–31.2% SL, while that in recently collected *A.melettinus* (N = 14) is 22.9–26.3, and the two species are easily distinguished by the deeper body of the former.

Our search for recent specimens of *A.melettinus* from the vicinity of Mumbai led to the examination of two specimens of *Amblypharyngodon*, BNHS 560–61, 44.5 and 56.8 mm SL, from Tilase on the Vaitarna River, Maharashtra, ca. 50 km from Mumbai. These have 66 and 67 scales in the lateral series, 25 and 27 scale rows between the dorsal- and pelvic-fin origins, and 31 and 35 circumpeduncular scales. Although these meristics are suggestive of *A.mola*, these specimens group with *A.melettinus* in morphological space (Figure 2B). We provisionally assign them to *A.mola* noting that Wahab et al. (2003) observed that *A.mola* is popularly used in aquaculture in India along with other species of fish and prawns, which may explain its presence in the Vaitarna. Wagh and Ghate (2002) too, recorded *A.mola* from Mula and Mutha rivers of Maharashtra.

### *Amblypharyngodon microlepis*

In differentiating *A.microlepis* from *A.melettinus*, Bleeker (1853: 141) relied primarily on the erroneous assumption that the lateral line was complete in the latter: “It has such a great resemblance to *Leuciscusmelettina*…”, observed Bleeker (1853), “that it might be kept to the same [species]. In *Leuciscusmelettina*, however, the lateral line goes up to the caudal fin, the scales are somewhat fewer and, at least according to the picture, the pectoral fins appear a little longer than the head.”

Bleeker was evidently misled by the illustration of *A.melettinus* that accompanied the original description (Valenciennes 1844: pl. 501, reproduced here as Figure 7C), which seems to show a complete lateral line. In the syntypes of *A.melettinus*, as noted also above, the lateral line extends only to the first 16–21 scales (Valenciennes did not provide a scale count in the original description); see also Figure 7A, which shows a 65.7 mm syntype (MNHN 3812), in which the lateral line consists of approximately 20 pored scales, terminating just posterior to the pelvic-fin origin.

Bleeker’s (1853) original description of *A.microlepis* contains some information of diagnostic value, including that the lateral line terminates just anterior to the tip of the pectoral fin, the lateral-line series consisting of 60 scales; that there were 20 scale-rows in transverse line on the body; and that the dorsal-fin origin lay between the pelvic-fin origin and anal-fin origin.

Vidthayanon and Kottelat (1990), however, examined four specimens from Bengal (RMNH 7043) identified as *A.microlepis* by Bleeker, reporting that these contained 58–65 scales in the lateral-line series, the first 8–12 of which were perforated; and had a body depth of 24.3–26.3% SL. The specimen AMS B7593, from Orissa, India, identified as *A.microlepis* by Day, has 55 scales in the lateral-line series. These data enable *A.microlepis* to be unambiguously distinguished from *A.grandisquamis*, which has 42–56 scales in lateral series and a body depth of 26.9–31.2% SL.

### *Amblypharyngodonmola* etc.

*Amblypharyngodongrandisquamis* is easily distinguished from *A.mola* by possessing fewer scales in the lateral series: 42–56, vs. 69–73 in *A.mola* from the putatively topotypical West Bengal specimens examined by us, 65–79 in the Orissa specimens examined by Vidthayanon and Kottelat (1990), and 65–75 reported by Day (1878: 555) from across the species’ range in India. Additionally, *A.grandisquamis* differs from *A.mola* by having a shorter dorsal fin, smaller eye, fewer circumpeduncular scales and fewer lateral scale rows (see Diagnosis).

There remain five additional nominal species of *Amblypharyngodon* reported from the Indian peninsula: *A.chakaiensis* Babu Rao and Nair (type locality the Chakai backwater near Trivandrum [Thiruvananthapuram], Kerala); *Leuciscuschitul* Sykes (‘Inderance River near Chakun, Deccan’); *Rhodeusindicus* Jerdon (Palghat [Palakkad] River, Kerala); *Brachygrammajerdonii* Day (Cochin [Kochi], Kerala); and *Rhodeusmacrocephalus* Jerdon (Cavery [Kaveri] River and Carnatic [Karnataka], southern India). Except for *A.jerdoni*, which Day (1878: 555) himself relegated to the synonymy of *A.melettinus*, none of these is represented by type material or accompanied by a description enabling differential diagnosis with congeners. Elucidation of their identity must necessarily await a revision based on fresh collections in India. We note, however, that Day (1878), who was last to review the Indian species of *Amblypharyngodon*, considered *A.indicus* and *A.jerdoni* to be synonyms of *A.melettinus*; and *A.chitul* and *A.macrocephalus* synonyms of *A.mola. Amblypharyngodonchakaiensis*, distinguished from *A.melettinus* by Babu and Nair (1978) in having 16 pectoral-fin rays and six scale-rows between the lateral line and pelvic fin base, has been treated as valid by some subsequent authors (e.g., Talwar and Jhingran 1991, Jayaram 2010) and as a synonym of *A.melettinus* by others (e.g., Menon 1999). No type material of *A.chakaiensis* is known, and an attempt in 1996 by one of us (RP) to recollect topotypes from the Aakulam ‘lake’ at Chacka, a suburb of Thiruvananthapuram, was abandoned owing to the high degree of aquatic pollution. In any event, *A.grandisquamis* can be unambiguously diagnosed from *A.chakaiensis* by having only 11–14 (14) branched pectoral-fin rays (vs. 16 in *A.chakaiensis*), although this by itself is hardly a character by which cyprinid species can be validated.

### Genetic analysis

While our genetic analysis clearly separates *A.grandisquamis* from its peninsular-Indian congeners *A.melettinus* and *A.mola*, it does not fully resolve the sister-group relationships of *A.grandisquamis*. Our COI and the COI+16s combined analyses recover *A.melettinus* as the sister group of [*A.grandisquamis* + *A.mola*], whereas our 16s phylogram recovers *A.mola* as the sister group of [*A.grandisquamis* + *A.melettinus*].

The uncorrected pairwise genetic distances for COI between *A.grandisquamis* and *A.melettinus*, *A.mola* and *A.chulabhornae*, of 9%, 6% and 10%, respectively, are consistent with the distances observed between independently validated species (Ward 2009), lending confidence to our consideration of *A.grandisquamis* as a valid species.

The non-significant results of the neutrality tests suggest that there has been no recent range expansion or population bottleneck in *A.grandisquamis* in Sri Lanka.

### Conservation

Jordan and Starks (1917) described the type locality of *A.grandisquamis* as “river at Colombo”. The only river that drains Colombo is the Kelani, which then should fix the type locality (Pethiyagoda 1991: 25). Though still recorded in some marshes and reservoirs in the suburbs of Colombo, *A.grandisquamis* is met with less frequently in the ‘wet zone’ (precipitation more than 2,000 mm/y) of the island’s southwestern quarter, within which Colombo lies, than in the ‘dry zone’ (precipitation less than 2,000 mm/y). There is no basis to the claim made by De Silva et al. (2015) that *A.grandisquamis* is restricted to the wet zone while *A.melettinus* is widely distributed in the dry zone.

The National Red List of Sri Lanka (MOE 2012) assesses *A.grandisquamis* as Endangered (presumably because it is endemic) and *A.melettinus* as Least Concern. There is, however, only a single species of *Amblypharyngodon* inhabiting in Sri Lanka, adding weight to the recommendation by Sudasinghe et al. (2018b) that conservation plans be predicated on sound taxonomy. *Amblypharyngodongrandisquamis* is exploited in the fishery and accounts for the highest biomass production among minor indigenous cyprinids in Sri Lanka (Pet et al. 1996). Nevertheless, given its wide distribution across the island’s lowlands, and its abundance in the dry zone, where inland fisheries are most intensive, *A.grandisquamis* is not a species of immediate conservation concern.

Bossuyt et al. (2004) showed that despite Sri Lanka having been terrestrially connected to southern peninsular India during glacial sea-level low-stands until the Holocene, endemism within several faunal groups, including freshwater fishes, is much higher on the island than had previously been suspected. This inspired authors to subject Sri Lankan fishes until then ascribed to ‘Indian’ species to review the identities of the island’s freshwater fishes through morphological and molecular comparisons with their Indian congeners, showing them in many cases to be insular endemics: e.g., *Mystuszeylanicus*Ng and Pethiyagoda (2013); *Pethiamelanomaculata* (Deraniyagala), Batuwita et al. (2015); *M.nanus*Sudasinghe et al. (2016); *Ompokceylonensis* (Günther) and *O.argestes*Sudasinghe and Meegaskumbura (2016); *Labeolankae* Deraniyagala and *L.heladiva*Sudasinghe et al. (2018a). *Amblypharyngodongrandisquamis* becomes the latest such species, and is unusual among Sri Lanka’s endemic freshwater fishes in that its range spans both principal climatic zones, a distribution otherwise observed in only three of the approximately 50 endemics now recognised in the island.

## Conclusions

Since the description of *A.grandisquamis* by Jordan and Starks (1917) from Sri Lanka, the identity of the species of *Amblypharyngodon* inhabiting the freshwaters of the island has been confused for most of the past century. Two species, *A.grandisquamis* and *A.melettinus*, had been frequently recognised in the Sri Lankan literature without, however, a specimen-based study. Here, by incorporating both morphological and molecular data, we conclude that only a single species of *Amblypharyngodon* (*A.grandisquamis*) occurs in the island, which is, morphologically as well as genetically distinct from the species of *Amblypharyngodon* in the Indian peninsula.

## Supplementary Material

XML Treatment for Amblypharyngodon grandisquamis
